# A neck-sparing short stem shows significantly lower blood loss in total hip arthroplasty compared to a neck-resecting short stem

**DOI:** 10.1038/s41598-023-47008-9

**Published:** 2023-11-11

**Authors:** Christian Stadler, Bernhard Schauer, Katja Brabec, Clemens Schopper, Tobias Gotterbarm, Matthias Luger

**Affiliations:** 1 Department for Orthopaedics and Traumatology, Kepler University Hospital GmbH, Med Campus III, Krankenhausstr. 9, 4020 Linz, Austria; 2 Johannes Kepler University Linz, Altenberger Str. 96, 4040 Linz, Austria

**Keywords:** Outcomes research, Bone

## Abstract

Short stems are associated with a significantly lower blood loss (BL) compared to straight stems in total hip arthroplasty (THA). Different types of stems differ in design, fixation and level of femoral neck osteotomy. Therefore, we sought to evaluate the difference regarding the perioperative BL between two short stems with different designs in direct anterior approach (DAA). A total of 187 THA performed by a single surgeon were analysed. 107 patients received a neck-resecting (Group A) and 80 patients a neck-sparing short stem (Group B). Blood counts of the day before surgery and of two days after surgery were evaluated. Total blood volume and BL were calculated. Additionally, duration of surgery was analysed. The perioperative BL was significantly lower in Group B (451.4 ± 188.4 ml) compared to Group A (546.6 ± 232.7 ml; *p* = 0.002). The postoperative haematocrit (31.6 ± 3.7% vs. 30.4 ± 4.4%; *p* = 0.049) and haemoglobin-level (11.0 ± 1.3 g/dL vs. 10.4 ± 1.5 g/dL; *p* = 0.002) were significantly higher in Group B. Duration of surgery was significantly shorter in Group B (62.0 ± 11.4 min vs. 72.6 ± 21.8 min; *p* < 0.001). The use of a neck-sparing short stem leads to a significantly decreased BL compared to a neck-resecting short stem in DAA THA. A less extensively conducted capsular release necessary for optimal femoral exposition might lead to a lower perioperative BL and shorter durations of surgery.

## Introduction

 Minimally invasive (MIS) approaches have been introduced in recent years in total hip arthroplasty (THA)^1^. Due to their geometry, standard straight stems require more exposition and resection of the proximal femur for an optimal component placement compared to modern generation short stems^2^. Additionally, short stems allow smaller skin incisions with less soft tissue damage, while still enabling an accurate reconstruction of the pre-arthritic hip-anatomy^3–5^. While short stems in general are quite heterogenous with different designs and fixation philosophies, some modern “calcar loading” short stems like the Optimys Stem (Mathys Ltd. Bettlach, Switzerland), the NANOS Stem (Smith&Nephew, Marl, Germany) or the ANA.NOVA proxy hip stem (ImplanTec GmbH, Moedling, Austria) aim for an individual restoration of pre-arthritic hip biomechanics with even lower bone loss using a femoral neck sparing design^6,7^. In comparison to neck-resecting short stems, the femoral neck osteotomy is conducted further proximally and can be slightly varied depending on the patient’s anatomy, which potentially allows for a more individual restoration of the proximal femur’s anatomy and a more physiological load distribution^8^. However, analysis of the individual anatomy of each patient and exact preoperative templating is crucial when performing THA using a neck-sparing short stem as the restoration of the pre-arthritic hip biomechanics strongly depends on a precisely conducted femoral neck osteotomy as it significantly influences stem position and consequently hip-biomechanics—especially the hip offset^6^.

Although the differentiation between the effect of the stem itself on the perioperative blood loss (BL) and the approach it is implanted through is difficult, the use of short stems also seems to enable lower levels of perioperative BL and lower rates of blood transfusions compared to the use of straight-stems^9^. Nonetheless, perioperative BL is inevitable when performing THA. Reports of overall BL associated with THA range from 540 to 1600 ml depending on different factors like approach, implant, duration of surgery and perioperative blood loss prophylaxis^9–11^.

With an increasing socio-economic burden on health care providers, rising numbers of outpatient THA and in general increasing trends towards early mobilization and early discharge from hospital, optimization of the perioperative management becomes more and more important^12–15^. One contributing factor to postoperative pain, swelling and possibly delayed mobilization is the perioperative BL and consecutive hematomas, which are to some extent inevitable after THA^16,17^. Furthermore, a lower perioperative BL might positively influence the overall outcome, as blood transfusions after THA seem to be associated with higher rates of periprosthetic infections, complications and longer length of stay at the hospital^18–20^.

Up to date there are hardly reports regarding differences in BL between short stems with different designs and different fixation philosophies requiring different levels of femoral neck resection. Therefore, we sought to evaluate possible impacts of stem design and level of femoral neck resection on the perioperative BL associated with THA.

## Materials and methods

### Study population

A consecutive series of 254 hips with unilateral index surgery between January 1st 2017 and July 31th 2022 operated by a single surgeon using a MIS direct anterior approach (DAA) to the hip were retrospectively screened for inclusion. The medical records until discharge from hospital were evaluated. In 159 of the cases the Fitmore hip stem (ZimmerBiomet, Warsaw, IN, USA) combined with the Allofit/-S press-fit acetabular cup (ZimmerBiomet, Warsaw, IN, USA) (Group A) and in 95 of the cases the ANA.NOVA proxy hip stem (ImplanTec GmbH, Moedling, Austria) combined with the ANA.NOVA Alpha acetabular cup (ImplanTec GmbH, Moedling, Austria) (Group B) were implanted.

**Figure 1:**
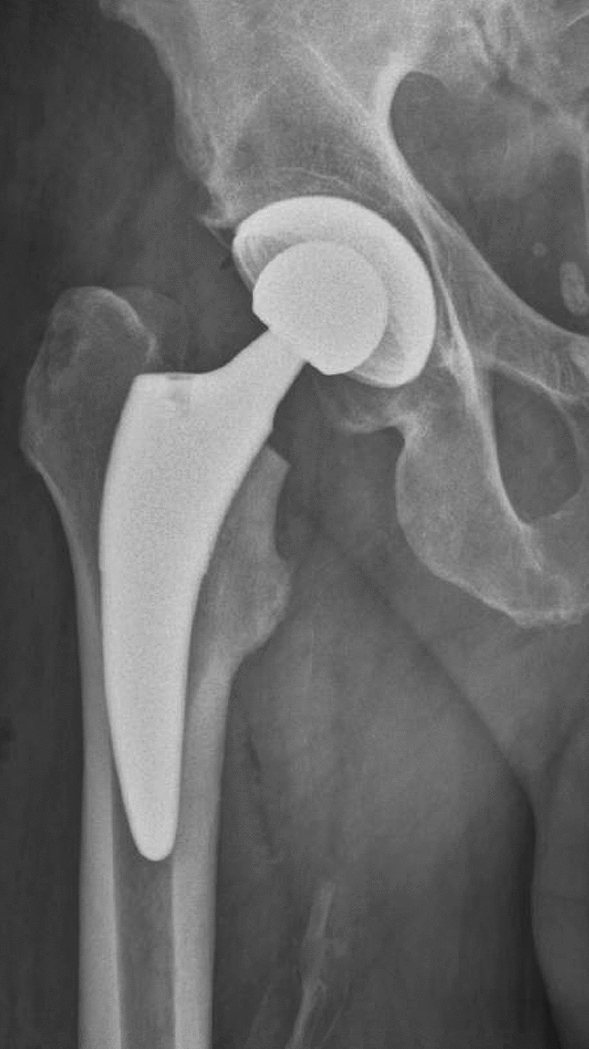
Shows the neck-resecting Fitmore hip stem and press-fit acetabular cup used within Group A.

The cementless titanium alloy (TiAl6V4) Fitmore hip stem features a porolock Ti-VPS coating in the proximal part for enhanced bone ingrowth. It is available in four different neck angle options and in 14 different sizes for each offset option^2,21^. To achieve press-fit fixation, the stem has a triple tapered design. It can be classified as neck-harming short stem according to the recommended level of resection of the femoral neck (Fig. 1)^21,22^. Reports available in the literature show excellent clinical performance of this stem with high patient satisfaction and high survival rates of 93.7% for revision for all causes and 99.6% for revision due to aseptic loosening at a follow up of 8.6 years^2,21,23^.

**Figure 2:**
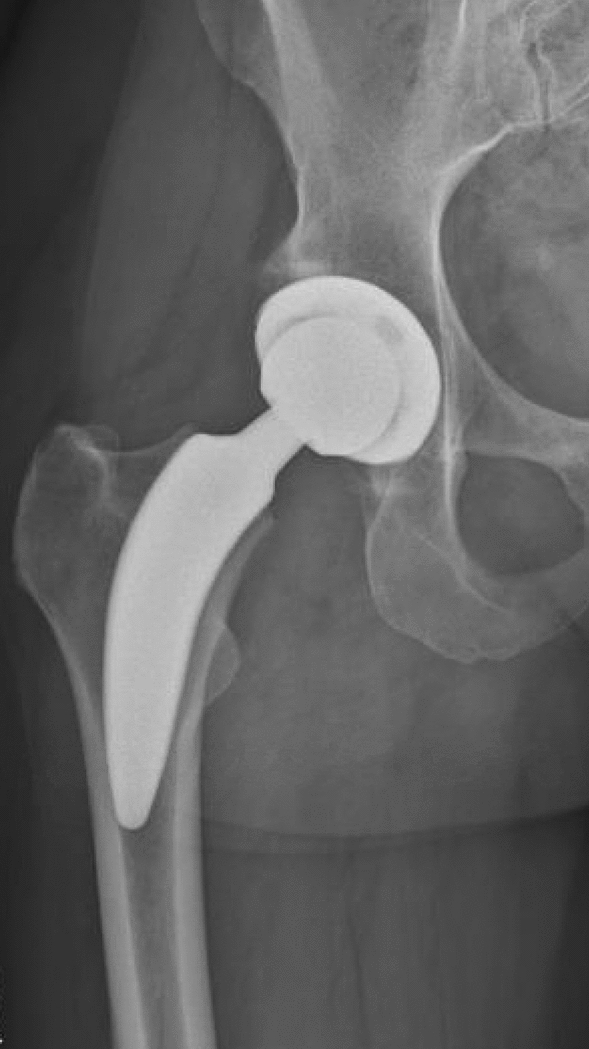
Shows the neck-sparing ANA.NOVA proxy hip stem and press-fit acetabular cup used within Group B.

The cementless titanium alloy (TiAl6V4) ANA.NOVA proxy hip stem has a rough titanium plasma coating with electrochemically applied hydroxyapatite (BONIT) to enhance osteointegration. It features a triple tapered design with a calcar guided press fit fixation with a 3-point anchorage with the main fixation zone between medial calcar and lateral cortex. It is available in 12 different sizes with two offset options for each size^24^. It can be classified as partial femoral neck-sparing short stem according to the recommended level of resection of the femoral neck (Fig. 2)^22^. Up to date, there are hardly reports regarding the clinical performance and survival rate of this stem. However, it seems to enable satisfying hip geometry restoration and low revision rates due to subsidence at a follow up of 3 years^24,25^.

The medical records of the patients were screened primarily for laboratory results regarding perioperative BL. Patients with systematic diseases affecting the blood count and lacking perioperative and postoperative laboratory results were excluded from this study. The first 50 cases performed via DAA in combination with the Fitmore hip stem were defined as learning curve for the approach itself and were therefore excluded from the study^26–28^. Further exclusion criteria were any other approaches to the hip apart from the DAA as well as the occurrence of any intraoperative complications such as fractures.

The study was approved by the ethics committee of the medical faculty of the Johannes Kepler University Linz (Reference number: 1140/2022). Due to the retrospective study design with evaluation of pre-existing medical records the need for informed consent was waived by the ethics committee of the medical faculty of the Johannes Kepler University Linz. All procedures performed were in accordance with the principles of the Declaration of Helsinki.

### Surgical technique and postoperative treatment protocol

In all cases the standardized peri- and postoperative protocol was identical. Tranexamic acid (20 mg per kilogram of body weight) was administered routinely prior to skin incision. Surgical procedures were performed by a single fellowship-trained consultant. In all cases a minimally invasive DAA without the use of a traction table was performed as previously described^29^. Components were implanted according to the manufacturer’s instructions and aiming for a restoration of the pre-arthritic biomechanics of the affected hip. Local infiltration anaesthesia (0.5 mg Epinephrin and 1 mg Ropivacaine per 100 ml Sodium chloride) was performed in all cases prior to wound closure. Weight-bearing was tolerated immediately after surgery. In case of a postoperative haemoglobin level below 8 g/dL, blood transfusion was conducted if clinical symptoms of anaemia were present. In case of a postoperative haemoglobin level below 7 g/dL, blood transfusion was conducted regardless of clinical symptoms of anaemia.

### Medical record evaluation and blood loss calculation

The medical record of each patient was screened for laboratory results in order to calculate the perioperative BL. The blood count of the day of admission to the surgical unit, which was scheduled one day prior to surgery, was analysed regarding Haematocrit- and Haemoglobin-Level. Laboratory results of the same parameters were analysed for the second day after surgery as well. The patients’ estimated blood volume (BV) was calculated using the formula described by Nadler et al.^30^: $$BV male patients=604+0.0003668 \times \left[{size\left(cm\right)}^{3}\right]+32.2 \times weight\left(kg\right);BV female patients=183+0.000356\times \left[{size\left(cm\right)}^{3}\right]+33\times weight\left(kg\right).$$ The estimated perioperative BL was calculated using a modified version of the formula described by Mercuriali et al.^31^ using the Haematocrit level measured on the second day after surgery instead of the Haematocrit level measured on the fifth day after surgery as described by Mercuriali et al.^31^ $$Estimated BL=BV \times {(Hct}_{preoperative}-{Hct}_{2 days postoperative})+ml of transfused blood$$. Blood transfusions within this time period were evaluated and taken into account when calculating the estimated BL. Additional parameters such as patient specific data like gender, age, height, weight, BMI and ASA-Score as well as surgery specific data like component sizes and duration of surgery and length of stay at the hospital were analysed too.

### Statistical analysis

Statistical analysis was performed using SPSS version 28 (IBM SPSS statistics, Chicago, IL, USA). Arithmetic mean value and standard deviation were calculated for metric scaled data. Kolmogorov–Smirnov-Test was performed to test for normal distribution. For normally distributed parameters Chi-Square-Test was performed to analyse categorial parameters while t-Test was performed to analyse metric scaled parameters. For non-normally distributed metric parameters Man-Whitney-U-Test was conducted. A *p* value < 0.05 was considered as statistically significant.

## Results

**Figure 3:**
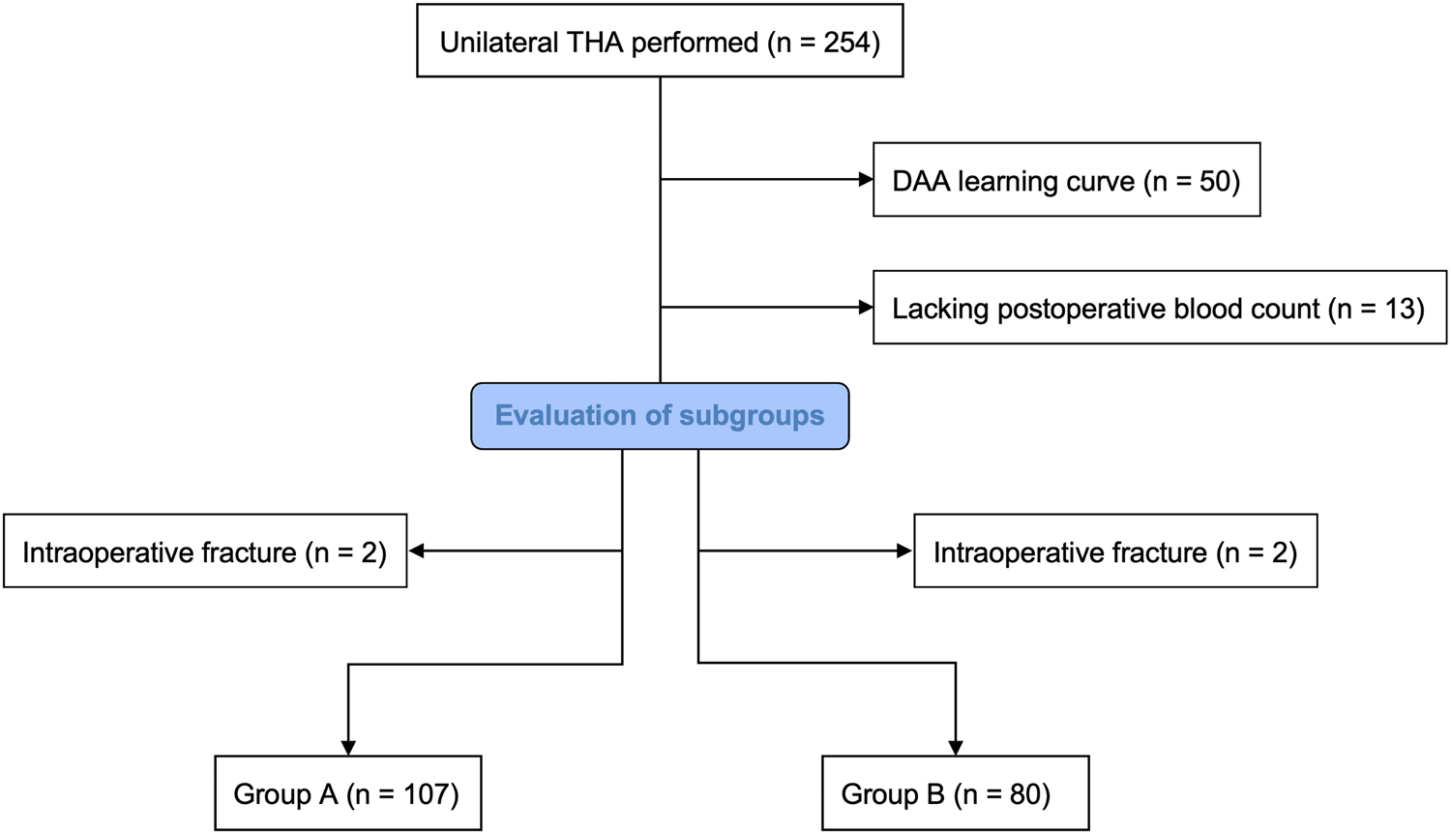
Shows the formation of the two study subgroups depending on the implant used for Total Hip Arthroplasty (THA) performed via Direct Anterior Approach (DAA); Group A: neck-resecting Fitmore hip stem; Group B: neck-sparing ANA.NOVA proxy hip stem.

In total 187 patients were included for analysis in the present study. A total of 4 intraoperative fractures with 2 cases in each study group (*p* = 0.574) occurred within the study population (Group A: 1 intraoperative acetabular fissure and 1 intraoperative fracture of the greater trochanter—both treated conservatively; Group B: 1 intraoperative femoral shaft fracture treated with a revision stem and cerclages and 1 intraoperative fracture of the greater trochanter treated conservatively) and were excluded from the analysis (Fig. 3). 52.9% of the study population were female patients and the mean age was 67.8 ± 10.7 years within the study population (Table 1). There were no significant differences regarding the preoperative calculated BV, Haematocrit- or Haemoglobin-levels between the two study groups (Table 2). Postoperatively, the calculated BL of Group B (451.4 ± 188.4 ml) was significantly lower compared to Group A (546.6 ± 232.7 ml; *p* = 0.002). The haematocrit (Group A: 30.4 ± 4.4%; Group B: 31.6 ± 3.7%; *p* = 0.049) and the haemoglobin-level (Group A: 10.4 ± 1.5 g/dL; Group B: 11.0 ± 1.3 g/dL; *p* = 0.001) at the second day after surgery were significantly lower within Group A. Blood transfusions were administered significantly less often in Group B (1.2%) compared to Group A (9.3%; *p* = 0.025). The average duration of the surgery was significantly shorter within Group B (62.0 ± 11.4 min) in relation to Group A (72.6 ± 21.8 min; *p* < 0.001). Overall length of stay at the hospital was significantly shorter within Group B (6.1 ± 1.7 days) compared to Group A (6.8 ± 2.9 days; *p* = 0.029).

**Table 1:** Shows the patient demographics of the study population.

Variable	Group A	Group B	Overall	*p* value
Number of patients	107	80	187	–
Age at operation (years)	67.2 ± 10.8	68.6 ± 10.6	67.8 ± 10.7	0.377
BMI (kg/m^2^)	27.5 ± 4.3	27.2 ± 4.3	27.4 ± 4.3	0.635
Height (cm)	169.7 ± 9.2	169.6 ± 9.5	169.6 ± 9.2	0.920
Weight (kg)	79.8 ± 16.0	78.7 ± 15.4	79.3 ± 15.7	0.626
Gender (female:male)	54:53	45:35	99:88	0.263
Side (left:right)	53:54	30:50	83:104	0.068

**Table 2:** Shows the results of the evaluation of the perioperative blood counts with the preoperative values from the day before surgery and the postoperative values from the second day after surgery (Hct = Haematocrit in %; Hb = Haemoglobin in g/dL).

Variable	Group A	Group B	Overall	*p* value
Preoperative Hct	41.3 ± 3.2	41.1 ± 3.3	41.2 ± 3.2	0.686
Preoperative Hb	14.1 ± 1.2	14.2 ± 1.3	14.2 ± 1.3	0.472
Total blood volume (ml)	4778.1 ± 887.4	4714.4 ± 883.3	4750.9 ± 883.9	0.627
Postoperative Hct	30.4 ± 4.4	31.6 ± 3.7	30.9 ± 4.1	0.049
Postoperative Hb	10.4 ± 1.5	11.0 ± 1.3	10.7 ± 1.5	0.002
Calculated blood loss	546.6 ± 232.7	451.4 ± 188.4	505.9 ± 219.5	0.002

## Discussion

The results of this study reveal a significantly lower BL of a partially neck-sparing short stem compared to a neck-resecting short stem. Additionally, the average duration of the surgery as well as the length of stay at the hospital were significantly shorter for THA using a neck-sparing stem with epi-metaphyseal fixation.

Up to date, there are hardly reports investigating the differences in perioperative BL between different types of hip stems. While previous studies showed lower rates of perioperative BL comparing short stems to straight stems implanted through different approaches, this is the first study investigating the differences in perioperative BL between two different types of short stems implanted by a single surgeon through the same standardized MIS DAA without traction table and the same perioperative treatment protocol for all patients^9^.

A total of 4 intraoperative fractures occurred within the study population with no significant difference between the two study groups (2 intraoperative fractures in each study group; *p* = 0.574). Those cases were excluded from the analysis to avoid distortion of the study’s findings as intraoperative fractures potentially significantly influence the perioperative BL as well as the duration of surgery. However, intraoperative and early postoperative femoral fractures are well-known complications associated with DAA^32^. The overall observed rate of 2.1% of intraoperative fractures within this study’s population is matching the findings of other reports^33–35^.

There are several factors influencing BL during hip surgery that must be considered. Within this study, a MIS DAA was performed in all cases, which seems to be associated with less BL compared to other approaches like a lateral or a posterior approach, which might be caused by a tendentially shorter skin incision and comparatively less soft tissue damage^36,37^. Tranexamic acid was administered routinely prior to skin incision by the anaesthetist within this study, which also seems to reduce BL following THA and also reduce the postoperative rate of blood transfusions after THA^38,39^. Additionally, local infiltration anaesthesia was performed in all patients prior to wound closure, which also seems to reduce BL after joint arthroplasty^40,41^. Within this study, no surgical drains were applied after THA, which seems to have benefits regarding minimizing the perioperative BL respectively the transfusion rate after THA as well^42,43^. In general, the calculated BL within this study is low compared to other reports investigating BL after THA via DAA^39^. This might be due to the modification of the formula described by Mercuriali et al.^31^ in form of evaluating the postoperative haematocrit level of the second day after surgery instead of the haematocrit level of the fifth day after surgery, as other reports evaluated blood counts taken at a later postoperative stage^9^. While still being mostly in line with other reports investigating the BL after THA within the first three postoperative days, the calculation of the absolute value of the perioperative BL after THA was less of an objective of this study than comparing the differences in perioperative BL between the two study groups^10^.

Duration of surgery also seems to be associated with BL during THA, which might be one contributing factor regarding the lower calculated BL within Group B of this study, as the duration of surgery was significantly lower in Group B compared to Group A^42^. However, the other main factor associated with lower BL within this study might be the partially femoral neck-sparing short stem with epi-metaphyseal fixation used within Group B. In general, short stems seem to enable THA with tendentially lower amounts of perioperatively BL when compared to straight stems^9^. In theory, the femoral stem used within Group B of this study combined with the DAA required a more proximally conducted femoral osteotomy due it’s fixation philosophy which tendentially led to a more upright angle of the surface of the osteotomy and therefore allowed an easier achievement of an optimal exposition of the femoral neck for implanting the stem. Additionally, the easier exposition of the proximal femur might have allowed for a less extensively conducted capsular release, which on one hand—due to the blood vessels surrounding the femoral neck—might have contributed to the lower BL within Group B and on the other hand also might have contributed to the on average significantly lower duration of surgery within this Group^44^.

Length of stay at the hospital on average was significantly shorter within patients who received the femoral stem with epi-metaphyseal fixation compared to those who received the stem with metaphyseal fixation (6.1 days vs. 6.8 days; *p* = 0.029). As for that matter, the perioperative BL consecutive hematoma and pain might have also had an influence on the length of stay at the hospital after THA, as other parameters like duration of the surgery, patient age or BMI seem not to necessarily influence length of stay at the hospital^45^.

However, there are some limitations to this study that must be kept in mind when interpreting the findings of this study. Firstly, this is a single centre retrospective cohort study with a single surgeon setting. Moreover, due to the design of the present study, no randomization was performed, as the surgeon chose which implant to use for each surgery. For example, patients with certain anatomical characteristics like Dorr-Type-C femora or valgus hips were less likely to receive a neck-sparing short stem within the first few cases, although over the further course of this study there was no contraindication for using a neck-sparing short stem due to certain anatomical characteristics with exception of severe hip dysplasia (Crowe > 1). Nevertheless, there is a chance of selection bias, which represents another limitation of this study. Additionally, estimated BL was calculated using the blood count of the second day after surgery, which also limits the results of this study, as blood counts from for example five days after surgery could have provided additional insights. However, a reasonable evaluation of postoperative blood counts other than on the second day after surgery were not possible within this study as many patients—to some extent caused by limits of capacity and infectiological reasons during the COVID-19 pandemic—were discharged from hospital before the fifth day after surgery without undergoing another blood sample. Therefore, evaluation of subsequent blood samples would have led to a considerably higher rate of patients lost to follow-up.

In summary, the use of a neck-sparing short stem leads to a significantly decreased BL in DAA compared to a neck-resecting short stem. A less extensively conducted capsular release necessary for optimal femoral exposition might lead to a lower perioperative BL with shorter durations of surgery. Therefore, the use of a neck sparing short stem can be recommended when performing DAA. However, further evaluations with bigger study populations are necessary to proof these findings.

## Data Availability

The datasets used and analyzed during the current study are available from the corresponding author on reasonable request.
